# Cross-sectional and Test-Retest Characterization of PET with [^18^F]FP-(+)-DTBZ for β Cell Mass Estimates in Diabetes

**DOI:** 10.1007/s11307-015-0888-7

**Published:** 2015-09-14

**Authors:** Matthew J. Freeby, Patricia Kringas, Robin S. Goland, Rudolph L. Leibel, Antonella Maffei, Chaitan Divgi, Masanori Ichise, Paul E. Harris

**Affiliations:** Department of Medicine, David Geffen School of Medicine, University of California, Los Angeles, CA 90404 USA; Naomi Berrie Diabetes Center, Columbia University Medical Center, New York, NY 10032 USA; Institute of Genetics and Biophysics Adriano Buzzati-Traverso, Consiglio Nazionale delle Ricerche, 80131 Naples, Italy; Division of Endocrinology, Department of Medicine, Columbia University Medical Center, New York, NY 10032 USA; Division of Nuclear Medicine and Kreitchman PET Center, Department of Radiology, Columbia University Medical Center, New York, NY 10032 USA

**Keywords:** Beta cell mass, Diabetes, VMAT2, [^18^F]FP-(+)-DTBZ, PET, Test-retest

## Abstract

**Purpose:**

The vesicular monoamine transporter, type 2 (VMAT2) is expressed by insulin producing β cells and was evaluated as a biomarker of β cell mass (BCM) by positron emission tomography (PET) with [^18^F]fluoropropyl-dihydrotetrabenazine ([^18^F]FP-(+)-DTBZ).

**Procedures:**

We evaluated the feasibility of longitudinal pancreatic PET VMAT2 quantification in the pancreas in two studies of healthy controls and patients with type 1 or 2 diabetes. VMAT2 binding potential (BP_ND_) was estimated voxelwise using a reference tissue method in a cross-sectional study, followed by assessment of reproducibility using a test-retest paradigm. Metabolic function was evaluated by stimulated c-peptide measurements.

**Results:**

Pancreatic BP_ND_ was significantly decreased in patients with type 1 diabetes relative to controls and the test-retest variability was 9.4 %.

**Conclusions:**

Pancreatic VMAT2 content is significantly reduced in long-term diabetes patients relative to controls and repeat scans are sufficiently reproducible to suggest the feasibility clinically VMAT2 measurements in longitudinal studies of new onset diabetes.

**Electronic supplementary material:**

The online version of this article (doi:10.1007/s11307-015-0888-7) contains supplementary material, which is available to authorized users.

## Introduction

Real-time molecular imaging has the potential to reveal the dynamics of β cell mass (BCM) in diabetes [1, 2]. We have hypothesized that the vesicular monoamine transporter, type 2 (VMAT2) might be a useful β cell target for longitudinal quantification by PET performed to obtain estimates of changes in BCM rather than absolute values [3–6]. Supporting this concept are reports documenting that (1) there exists an approximate five-fold natural variation in beta cell mass among healthy individuals [7]; (2) within the pancreas, VMAT2 co-localizes with insulin in β cells as determined by immunohistochemistry [8, 9]; (3) the pancreatic expression of VMAT2 closely parallels β cell insulin immunoreactivity in diabetic disease [10]; (4) there is a loss of VMAT2 signal paralleling the development of hyperglycemia and β cell loss in preclinical PET studies using rodent models of diabetes [3, 4, 11, 12]; (5) dihydrotetrabenazine (DTBZ)-based positron emission tomography (PET) radiotracers bind to VMAT2 with high affinity (i.e., Kd in the subnanomolar range) [5, 12–17] and selectivity [5, 18]; and (6) DTBZ-based PET tracers are useful in longitudinal studies requiring VMAT2 quantification in the central nervous system [19]. Additionally, we previously reported [^11^C]dihydrotetrabenazine ([^11^C]DTBZ) pancreatic PET uptake in humans with or without long-standing type 1 diabetes mellitus (T1DM) [20]. In this latter study, pancreatic binding of [^11^C]DTBZ was significantly reduced in subjects with long-standing T1DM compared to healthy controls.

More recently, researchers developed [18F]fluoropropyl-dihydrotetrabenazine ([^18^F]FP-(+)-DTBZ), which by virtue of its 120-min half-life and higher affinity binding to VMAT2 [13, 15], provides very high-quality PET images [21], allowing more accurate VMAT2 quantification relative to [^11^C]DTBZ. In this report, we document the results of a cross-sectional study of pancreatic VMAT2 in healthy controls and patients with T1DM and type 2 diabetes mellitus (T2DM) using PET with [^18^F]FP-(+)-DTBZ to estimate pancreatic BCM. To our knowledge, this is the first study evaluating VMAT2 in T2DM. Additionally, this study is the first to describe the reproducibility of pancreatic [^18^F]FP-(+)-DTBZ uptake measurements in human pancreata.

## Materials and Methods

This PET study was performed under the supervision of the Columbia University Medical Center Institutional Review Board and in accordance with the precepts established by the Helsinki Declaration. Our study was divided into two separate arms. The first arm evaluated [^18^F]FP-(+)-DTBZ pancreatic PET uptake in healthy controls and patients with T1DM or T2DM. The second arm evaluated the test-retest variability of VMAT2 quantification by PET scans with [^18^F]FP-(+)-DTBZ. In this segment, healthy controls and patients with T1DM underwent a second PET scan within 4 weeks of the first.

### Subjects

Twenty-five subjects completed the study; this included 14 healthy controls, 8 subjects with long-standing T1DM, and 3 subjects with T2DM. Healthy control subjects were excluded if fasting glucose levels were greater than 100 mg/dl or if there was a first-degree relative with T2DM. Subjects with diabetes were required to have been diagnosed at least 5 years prior to enrollment. Patients with diabetes were excluded if microvascular complications were present. Demographic and laboratory data are summarized in Table 1.

**Table 1:** Demographic and laboratory data

Parameter	Controls (*n* = 14)^a^	T1DM (*n* = 8)^b^	T2DM (*n* = 3)
Age (years)	27.5 ± 1.4	29.4 ± 4.3	55.0 ± 3.5*
Gender (male/female)	9:5	4:4	2:1
Weight (kg)	70.5 ± 2.7	75.2 ± 4.0	95.8 ± 10.9*
Body mass index (kg/m^2^)	23.5 ± 0.5	25.3 ± 0.9	31.4 ± 0.3*
Hemoglobin A1c (%)	5.0 ± 0.1	7.1 ± 0.2**	7.0 ± 0.1*
Insulin dose/day (units/kg/day)	N/A	0.57 ± 0.05	1.24^c^
Duration of diabetes (years)	N/A	18.5 ± 2.8	11.7 ± 2.4
Estimated glomerular filtration rate—Modification of Diet in Renal Disease Study formula (ml/min/1.73 m^2^)	100.7 ± 5.4	100.0 ± 4.1	90.0 ± 8.9

*N*/*A* not applicable

^a^Controls: *n* = 9 for cross-sectional study and *n* = 5 for test-retest study

^b^T1DM patients: *n* = 6 for cross-sectional study and *n* = 2 for test-retest study

^c^1 of 3 with T2DM requiring insulin

*p < 0.01, control vs. T2DM

**p < 0.01, control vs. T1DM

### Metabolic Testing

All subjects underwent mixed meal tolerance testing (MMTT). A 2-h MMTT was used to assess insulin production capacity, an indirect marker of BCM. Prior to undergoing MMTT testing, patients fasted for at least 6 h. Insulin was discontinued in all subjects with diabetes prior to testing; glucose levels ranged between 70 and 200 mg/dl before study start. No insulin was administered within 2 h of testing. In those using continuous insulin infusion therapies, basal rates were suspended 30 min prior to testing. Two-hour MMTT was performed as per previous published reports [20, 22]. Venous serial samples were assayed for glucose (mg/dl), insulin (μIU/ml), and c-peptide (ng/ml) by previously described standard methods [20]. The peak c-peptide and area under the curve (AUC) c-peptide values were calculated as previously reported [20].

### PET Radiotracer

[^18^F]FP-(+)-DTBZ was synthesized as described previously [12]. In the first part of the study, [^18^F]FP-(+)-DTBZ was synthesized by AVID Radiopharmaceuticals Ltd. (Philadelphia, PA). [^18^F]FP-(+)-DTBZ was synthesized at the Kreitchman PET center of Columbia University in the second part of study, which included the test-retest segment.

### PET Scans

All subjects were studied after an overnight fast. Type 1 diabetic subjects were instructed to not take their usual morning insulin dose. Patient blood glucose was monitored before and during the scan and insulin dosages were adjusted as needed to maintain glucose values between 70 and 200 mg/dl. Heart rate, blood pressure, and electrocardiogram were monitored continuously throughout the imaging procedure. Subjects were scanned lying supine on a Biograph mCT PET/CT camera (Siemens Medical Solutions USA, Inc., Malvern, PA) with reconstructed spatial resolution of approximately 6 mm. Data acquisition occurred over a period of up to 180 min. A low-dose abdominal computerized tomography (CT) scan, guided by the CARE Dose4D program (120 kV, 50–350 mA), was performed at the beginning of each session for the collection of attenuation mapping and anatomical data (matrix size = 512 × 512 × 74 and voxel size = 0.98 × 0.98 × 3.0 mm). Dynamic PET measurements were acquired in list mode over an axial field of that covered 22 cm beginning near the xiphoid process and extending caudally over the abdomen. [^18^F]FP-(+)-DTBZ (mean dose of 333 ± 44 MBq) was administered as a bolus over 2–3 min immediately after the start of the scan. The mean specific activity of [^18^F]FP-(+)-DTBZ at the time of injection was 158 ± 45 MBq nmol^−1^. There were no significant differences between control and patients with diabetes in injected radioactivity or mass of tracer. List mode-acquired PET data were reconstructed into 22 (2 h) or 28 (3 h) dynamic frames with increasing frame duration using a Hann-filtered back-projection algorithm with CT-based attenuation, time-of-flight scatter, random, scanner dead time, detector normalization, and radioactive decay corrections as provided by the manufacturer’s software. The final reconstructed images had a matrix size of 168 × 168 × 74 and a voxel size of 4.07 × 4.07 × 3.0 mm. For the test-retest studies, PET scans were performed as described earlier with the exception of extending the total scanning period to 180 min and subjects returned within 4 weeks for a second scan.

### Quantification of Pancreatic VMAT2 Binding

**Fig. 1:**
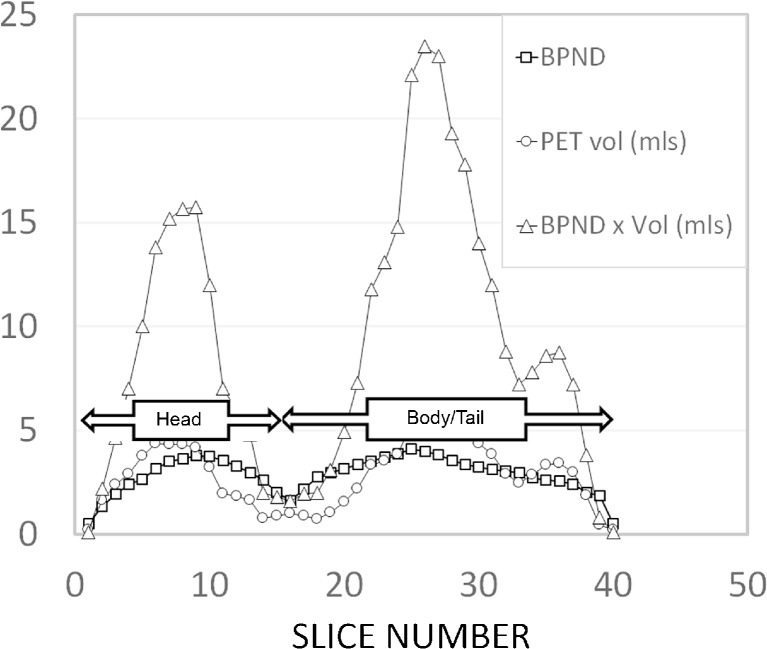
Sagittal slice by slice analysis of pancreatic VMAT2 binding obtained by parametric mapping of BP_ND_. A representative slice by slice analysis of VMAT2 binding in a healthy control pancreas, represented as the total volume of voxels with a BP_ND_ > 0.1 (*circles*), the average BP_ND_ of those voxels (*squares*) and the product of the voxel volume and mean BP_ND_ of the slice (*triangles*). *Y axis*; since BP_ND_ is dimensionless, there are no units on this axis. *X*-*axis*, the corresponding slice number starting at the head of the pancreas ending at the tail. The demarcation of head and combined body-tail of the pancreas was taken as the first local minima (*from left to right*) of mean BP_ND_ × total voxel volume.

The rationale and validity of applying voxelwise reference tissue, kinetic model-based quantification of pancreas VMAT2 by PET with [^11^C]DTBZ or [^18^F]FP-(+)-DTBZ has been previously documented [2, 5, 20, 21]. VMAT2 binding data analysis was performed using PET data analysis software by PMOD (PMOD Technologies LTD., version 3.5, Zurich, Switzerland). Reconstructed abdominal CT images were manually realigned if needed to correct for any visually detectable deviation from the anatomical position of the abdomen in three orthogonal planes. This individualized realignment transformation matrix was applied to dynamic PET data to ensure abdominal PET images were aligned in anatomical position. Then, abdominal CT images were coregistered to the summed realigned PET images over the initial 30 min for each subject. On the fused CT and summed PET images through multiple slices on the transaxial plane, volumes of interest were manually placed of over the spleen (approximately 130 cm^3^ in size). Time-activity curves (TACs) of the spleen were obtained by applying this volume-of-interest (VOI) on the dynamic PET images. Using the spleen as reference tissue input, voxelwise parametric images of VMAT2 binding potential, BP_ND_, which is linearly proportional to the VMAT2 density (B_avail_), were generated by using the reference tissue model, MRTM_O_ in PMOD [20, 23]. On the sagittal slices of BP_ND_ parametric images coregistered on the sagittal CT images, VOIs were placed over the pancreas using a threshold value of BD_ND_ > 0.1 and mean BP_ND_ values for each sagittal slice of the pancreas were plotted allowing a clear delineation of the head and combined body-tail of the pancreas (Fig. 1). Using this information, we calculated the PET region-of-interest (ROI) volume (total and combined body and tail), mean BP_ND_ of the whole pancreas, and mean BP_ND_ for the combined body and tail of the pancreas (Table 2).

**Table 2:** VMAT2 binding estimates with accompanying β cell function (stimulated AUC c-peptide and peak c-peptide) measurements

Controls-cross-sectional	β cell function		Pancreas body and tail^a^		
	AUC^b^	Peak^c^	PET ROI volume (ml)	Mean BP_ND_	BP_ND_ × volume (ml)^d^
Subject study number					
3001	347	4.9	50.5	1.97	99
3002	550	5.6	38.3	1.65	63
3004	557	7.7	83.3	2.92	243
3005	463	4.9	42.1	2.4	101
3006	372	4.1	53.5	1.79	96
3007	1056	14.0	49.7	2.25	112
3008	572	7.7	48.3	2.45	118
3009	558	6.4	43.5	2.12	92
3010	864	11.6	30.2	1.84	56
Controls-test-retest					
4001 test^e^	57	0.79			
4001 retest			22.4	2.644	59.4
4002 test	268	4.4	29.7	2.93	87
4002 retest			32.4	3.08	100
4004 test	407	9.0	32.3	3.18	103
4004 retest			27.6	2.95	81
4005 test	313	8.3	34.3	2.99	103
4005 retest			28.4	2.71	77
4006 test	104	1.2	27.0	2.67	72
4006 retest			30.7	2.97	91
T1DM-cross-sectional					
T1D-3001	0.50	0.1	24.3	1.17	29
T1D-3002	0.50	0.1	27.5	1.43	39
T1D-3004	0.50	0.1	26.5	1.85	49
T1D-3005	0.50	0.1	16.3	1.32	22
T1D-3006	0.50	0.1	32.3	1.09	35
T1D-3007	0.5	0.1	14.5	1.86	27
T1DM-test-retest					
T1D-4007 test	68	0.74	13.2	2.22	29
T1D-4007 retest			12.3	2.62	32
T1D-4008 test	49	.64	22.8	3.09	71
T1D-4008 retest			24.5	2.89	71
T2DM-cross-sectional					
T2D-3001	393	4.52	19.2	1.66	32
T2D-3002	623	4.06	36.2	2.76	100
T2D-3003	1128	9.06	35.4	1.37	49

^a^As defined in Fig. 1

^b^AUC insulin c-peptide (ng/ml × minutes)

^c^Peak insulin c-peptide concentration (ng/ml) taken from highest serum value from the serial blood draws following the mixed meal stimulus (MMTT).

^d^Also referred to in the text as the functional binding capacity

^e^Subject did not successfully complete the first scan. These data used only in Fig. 3

### VMAT2 Binding Comparisons

Previously, we documented that VMAT2 expression more closely paralleled beta cell insulin expression in the body and tail of the pancreas [8]. The PET-estimated VMAT2 binding in the pancreas was compared between patient and control groups in two ways. The first comparison metric was the mean BP_ND_ value calculated over the combined body and tail of the pancreas. To better estimate the total beta cell mass, we calculated a second metric, the functional binding capacity (FBC) [20]. The FBC represents the sum of all individual pancreatic voxel BP_ND_ values greater than 0.1 multiplied by the total voxel volume (i.e., PET ROI volume) of the combined body-tail of the pancreas. These metrics were calculated for each subject and the group means calculated and compared.

### VMAT2 and Insulin Expression in Human Pancreatic Tissues

#### Islets, Exocrine Tissue, and Preparation of Total RNA

The islet and exocrine pancreas tissue used in these studies were historical samples obtained with institution review board approval from the New York Regional Islet Cell Resource Center at New York Presbyterian Hospital, Columbia Presbyterian Campus. The isolation of islets and exocrine tissue and preparation of total RNA was previously described [6]. Human pancreas total RNA indicated as Pancreas 060 (Catalog Item 540024, LOT 06046024) was obtained from Agilent Technologies (Santa Clara, CA). Human pancreas total RNA, from a different individual, indicated as Pancreas 141 (Catalog Item 636577, LOT 1412464A) was been obtained from Clontech Laboratories, Inc. (Mountain View, CA). Total RNA from islets or purified acinar tissue was isolated using the RNA RNeasy Lipid Tissue Mini Kit (QIAGEN, Valencia, CA).

#### Quantitative Analysis of VMAT2 and INS Transcripts by RT-PCR

First-strand cDNA synthesis using 400 ng of total RNA as template was obtained using the QuantiTect Reverse Transcription Kit (QIAGEN, Valencia, CA). PCRs executed to measure the accumulation of specific transcripts were performed using as template the amount of cDNA obtained retro-transcribing 10 ng of RNA for β actin (ACTB) and Insulin (INS) and 40 ng of RNA for VMAT2. The QuantiTect SYBR Green PCR Kit (QIAGEN) was used to perform all the real-time quantitative PCR assays with annealing temperatures of 55–60 °C and extension times of 30–45 s, depending on the couple of primers used. Accumulation of specific transcripts was measured by real-time PCR, using the SmartCycler System (Cepheid, Sunnyvale, CA). Quantitative RT-PCR (qRT-PCR) reagent controls (reagents without any template or with 40 ng non-retro-transcribed RNA) were included in all the assays. Each assay was run in triplicate and independently repeated at least three times to verify the results; the mean of all measurement has been used for analysis and comparisons. The relative amount of specific transcripts was calculated by the comparative cycle threshold method given by Schmittgen and Livak [24]. To correct for sample to sample variations in quantitative RT-PCR efficiency and errors in sample quantitation, the level of ACTB transcripts was measured and used in normalization of specific RNA levels. All the value were made relative to one of the pancreata and then expressed as relative to the average of the relative values of the two pancreases. PrimeSTAR GXL DNA Polymerase from Clontech Laboratories, Inc. (Mountain View, CA) was used for semiquantitative PCR assays at the conditions recommended by the manufacturer (supplemental results). Based on the results of the real-time PCR, the program for the amplification of ACTB and INS was set on 30 cycles and the program for the amplification of VMAT2 was on 36 cycles, with annealing temperatures of 56–60 °C and extension times of 30–45 s, depending on the couple of primers used. The custom DNA oligos: V2-5_F (5′-CGGAAGCTCATCCTGTTCAT-3′) and V2-5_R (5′-TCTGAGATGGAGGCAGTGTG-3′) specific for Human VMAT2, as well IN-F (5′-CCGCAGCCTTTGTGAACC-3′) and IN-R (5′-GCTGGTAGAGGGAGCAGATG-3′) specific for Human INS, were synthesized by Life Technologies (Grand Island, NY). ACTB primers, specific for human β-actin, were obtained from QIAGEN (Hs_ACTB_1_SG QuantiTect Primer Assay Cat No QT00095431).

### Statistical Analysis

Descriptive statistics include the arithmetic mean and standard errors of the mean (S.E.M.). Least-squares linear regression was used to determine the correlation between outcome measures. The strength of the correlation is expressed in terms of *r*^2^. The significance of the correlation was calculated using the *t* distribution. Student’s *t* testing was used to assess the significance of the difference between means. All *p* values are 2-tailed. Test-retest reproducibility of PET ROI volume, BP_ND_, and BP_ND_ × PET ROI volume was assessed by calculating variability and reliability. The within-subject variability was defined as the absolute value of the difference between test and retest measurements expressed as a percentage of mean value of test and retest measurements. As metric of reliability we calculated the intraclass correlation coefficients (ICC) as previously described [25] using the Shrout and Fleis model 3,1 [26] according to the following formula.

$$ \mathrm{I}\mathrm{C}\mathrm{C}=\frac{\mathrm{MSb}-\mathrm{M}\mathrm{S}\mathrm{w}}{\mathrm{MSb}+\left(k-1\right)\mathrm{M}\mathrm{S}\mathrm{w}} $$

Where MSb and MSw are the mean sum of squares between and within subjects, respectively, and *k* is the number of trials or within-subject measurements (i.e., two in the present study). The coefficient value ranges from −1 (no reliability) to 1 (maximum reliability). The standard error of measurement (SEMt) and minimal detectable change (MDC) at the 95 % confidence interval were estimated as discussed by Weir [27] using the formulas

$$ \mathrm{SEMt}=\mathrm{S}\mathrm{D}\sqrt{1-\mathrm{I}\mathrm{C}\mathrm{C}} $$

$$ \mathrm{M}\mathrm{D}\mathrm{C}=\mathrm{SEMt}\times 1.96\times \sqrt{2} $$

For SEMt calculations, the standard deviation of the biases (i.e., test-retest) was used.

## Results

### Measures of β cell Function

Mixed meal tolerance testing assessed insulin secretion capacity, which is also a surrogate marker of BCM. AUC c-peptide and peak c-peptide levels from mixed meal tolerance testing are reported in Table 2. As expected, AUC c-peptide and peak c-peptide levels were significantly lower in patients with long-standing T1DM as compared to controls (*p* < 0.001). On average, AUC c-peptide for patients with type 1 diabetes was approximately 3 % the level seen in healthy controls. Six of eight patients with T1DM had no detectable c-peptide on mixed meal stimulation. AUC c-peptide levels in subjects with T2DM were not significantly different when compared to healthy controls (*p* = 0.18).

### Estimation of VMAT2 Binding in Patients and Controls

**Fig. 2:**
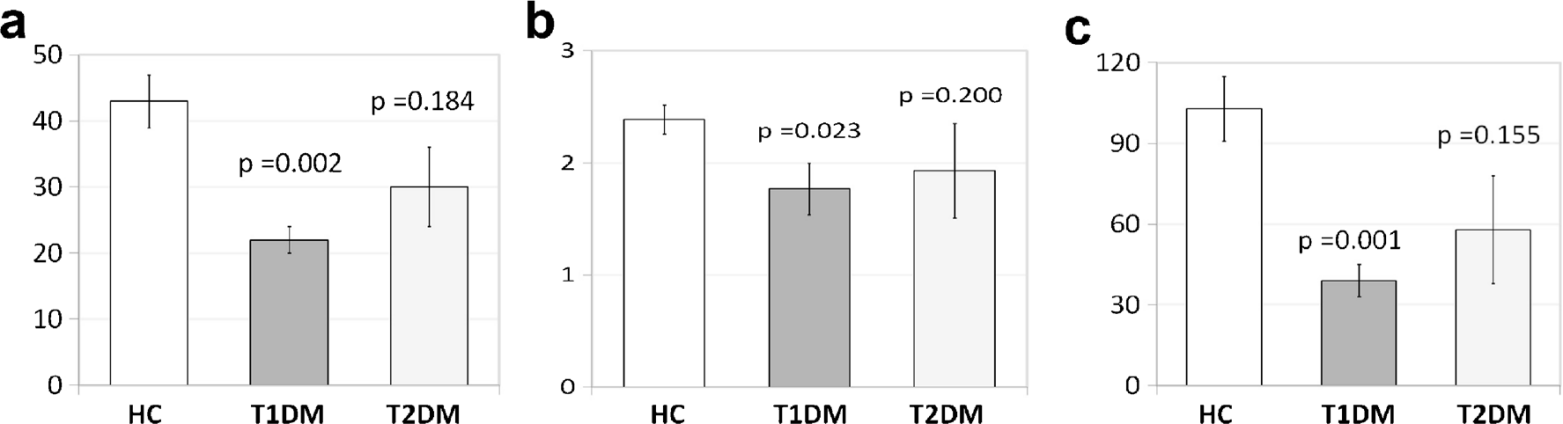
[^18^F]FP-(+)-DTBZ binding is reduced in the body-tail of the pancreas in T1DM patients vs. healthy control subjects. **a** The PET ROI volume, defined as the sum of the volumes of all voxels with a BP_ND_ > 0.1 contained with the combined body-tail of the pancreas was significantly lower in pancreas of T1DM patients (*n* = 8) relative to controls (*n* = 14). No significant difference between the PET ROI volume of patients with T2DM and controls was found, but the mean value of the PET ROI volume T2DM trended lower. **b** Pancreatic combined body-tail BP_ND_, which reflects tracer-specific binding relative to the VMAT2 poor reference region (Spleen), was estimated in healthy controls and patients with T1DM or T2DM. The mean BP_ND_ was significantly reduced by 26 % in pancreas of T1DM relative to controls. The mean BP_ND_ was not significantly different in the pancreas of T2DM patients but trended to lower than the values measured in controls. **c** The functional binding capacity (FBC) or the product of mean BP_*ND*_ and the PET determined voxel volume for the region was significantly lower (by 63 %) in pancreas of T1DM patients relative to healthy subjects. The FBC of patients with T2DM was not significantly different but trended to be lower relative to controls.

**Fig. 3:**
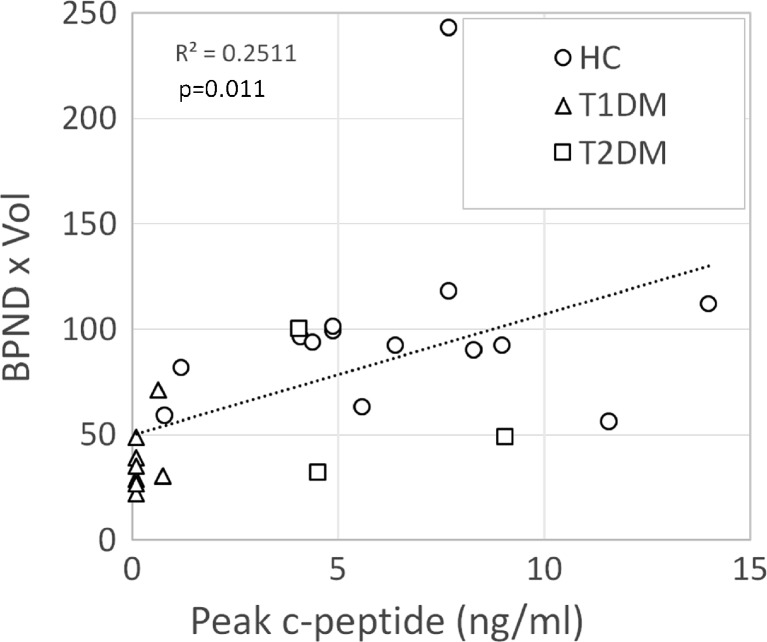
Association between binding potential and glucose-stimulated insulin secretion in controls and patients with T1DM or T2DM. BP_ND_ values and Peak c-peptide measures for each subject was evaluated for strength of association by linear correlation.

Pancreatic VMAT2 binding was compared among patients and controls. Previous post mortem analysis suggested VMAT2 binding is most specific to β cells in the body and tail of the pancreas. Therefore, we report pancreatic BP_ND_ in the body and tail alone (Table 3). Mean BP_ND_ in the pancreatic body and tail was decreased in patients with T1DM (1.77 ± 0.23) to 74 % of healthy control values (2.39 ± 0.13) (*p* = 0.023). The functional binding capacity in the body and tail (i.e., BP_ND_ × PET ROI Volume) was decreased in patients with T1DM (38 ± 6 ml) to 37 % of control subjects (103 ± 14 ml) (*p* = 0.001). Mean body and tail pancreatic BP_ND_ and BP_ND_ × PET ROI volume levels in patients with T2DM trended lower but were not significantly different from controls (Fig. 2). We found a significant correlation between peak c-peptide and functional binding capacity for the entire study population (Fig. 3).

**Table 3:** Summary statistics for combined cross-sectional and test-retest studies

	β cell function		Pancreas body and tail		
Cross-sectional study	AUC (% control)	Peak (% control)	PET ROI volume (ml) (% control)	Mean BP_ND_ (% control)	BP_ND_ × Volume (ml) (% control)
Healthy Controls					
Mean	463.43	6.47	43.12	2.39	102.85
S.E.M.	71.44	0.97	4.01	0.13	12.14
T1DM patients					
Mean	15.00 (3.2 %)	0.25 (3.8 %)	22.23 (52 %)	1.77 (74 %)	37.81 (37 %)
S.E.M.	9.66	0.10	2.46	0.23	5.56
T2DM patients					
Mean	714.67 (154 %)	5.88 (91 %)	30.27 (70 %)	1.93 (81 %)	60.33 (59 %)
S.E.M.	217.07	1.60	5.54	0.42	20.43
Student’s *t* test (2 tailed) *p* value of difference					
Control vs. T1DM	0.0001	0.0001	0.001	0.022	0.001
Control vs. T2DM	0.18	0.80	0.18	0.20	0.15
Test-retest study					
Mean variability			11.70 %	9.40 %	16.60 %
ICC			0.93	0.64	0.88
Standard error of measurement (SEMt) (via SD and ICC)			1.03	0.18	6.48
Minimal detectable change (MDC) (95 %CI) (% control)			2.85 (7 %)	0.49 (20 %)	17.96 (18 %)

### Test-Retest Characteristics

[^18^F]FP-(+)-DTBZ pancreatic imaging assessing variability and reliability was also performed. Two scans were completed in five healthy control subjects and two patients with T1DM. Repeat scans were performed less than 1 month after initial scanning. For measurements of BP_ND_ and BP_ND_ × volume, the mean variability was 9.4 % (range = 5.0 to 16.5 %) and 16.6 % (range = 0.0 to 28 %), respectively. The ICC reliability scores for the metrics examined ranged from 0.64 to 0.93) (Table 3).

### Measurement of Relative Expression Levels of VMAT2 and Insulin in Pancreas Tissue Samples

**Fig. 4:**
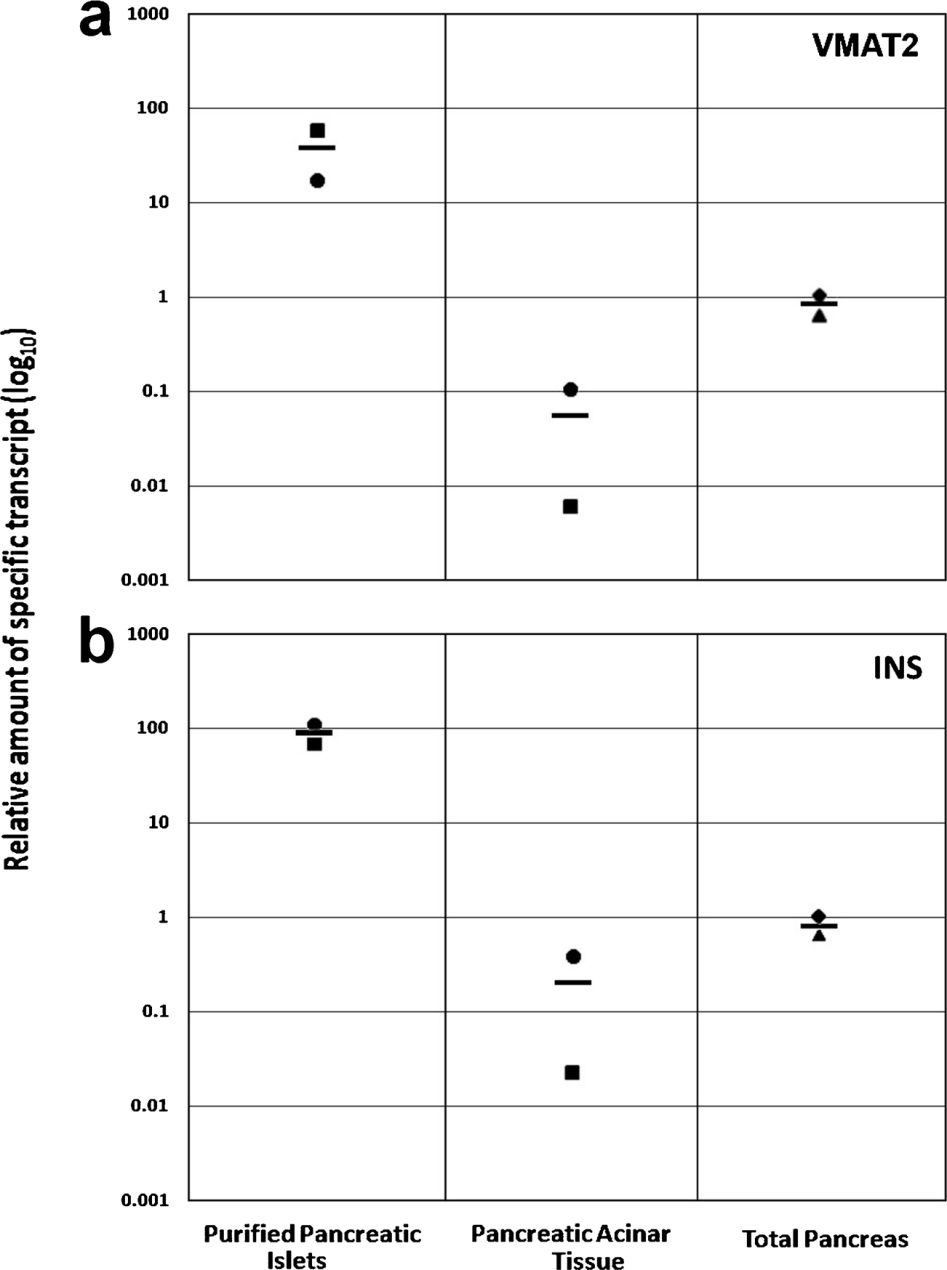
Measurements of relative levels of VMAT2 and insulin-specific transcripts in purified cadaveric islets, exocrine, and whole pancreas tissue. The relative level of **a** VMAT2or **b** Insulin expression was measured in the indicated tissue. The relative expression levels of VMAT2 or insulin message in the indicated matched samples (Islets 527 (*squares*), Islets 602 (*circles*), purified exocrine 527 (*squares*), purified exocrine 602 (*circles*) and unmatched whole pancreas (*triangles*) (human pancreas total RNA, LOT 1412464A Clontech) were normalized to one sample of unmatched whole pancreas (*diamonds*) (human pancreas total RNA, LOT 06046024 Agilent) and the average relative expression indicated by the *black bar*.

Quantification of VMAT2-specific transcripts revealed that islets express an average of greater than 500-fold more VMAT2 message than the corresponding exocrine tissue isolated from the same pancreas. The average fold difference of expression of VMAT2 in purified islets relative to whole pancreas was about 40-fold. Quantification of insulin-specific transcripts revealed a similar fold increase of insulin message expression in islets relative to exocrine tissue (Fig. 4). The average fold difference of expression of Insulin expression in purified islets relative to whole pancreas was about 80-fold.

## Discussion

The generally accepted view of the evolution of T1DM predicts declining BCM beginning in a prodromal period through diagnosis of disease and ending in the absolute dependence on exogenous insulin for glucose homeostasis [28]. In long-standing T1DM disease (>5 years), there is generally a near complete loss of the ability to secrete insulin [29]. In contrast, the pancreas in T2DM shows variable rates of β cell loss [30]. Past natural history studies of T1DM or T2DM or interventional trials have relied on limited autopsy samples or on multiple non-invasive or minimally invasive assays to estimate BCM. A shared characteristic of these latter assays is that they are surrogates, substituting measures of β cell function for anatomical BCM. These broadly adopted biomarkers of endocrine pancreas function (a.k.a. metabolic tests) include blood glucose concentration measurements, stimulated insulin secretion rates, serum insulin concentrations, HbA1c, and islet/insulin autoantibodies. These functional BCM biomarkers reflect the complex multifactorial phenotype of glucose sensing, insulin secretion, and insulin action. As such, they do not routinely assess potential β cell reserve capacity [31] or functional impairments due to β cell immaturity, dedifferentiation, distress, or inflammation. Quantitative measures of a simpler β cell phenotype (e.g., expression of single β cell restricted surface receptor) may provide a more accurate estimate of anatomical BCM. *In vivo*, clinical BCM monitoring is currently inadequate [32] because it does not reflect BCM in real time, requires large study populations because of the natural variability of BCM in humans, reflects confounding comorbidities and/or often occur too late (or too early) in the disease process to be useful sentinels of declining anatomical BCM. In this study, we have continued our evaluation of PET-based measurements of pancreatic [^18^F]FP-(+)-TBZ uptake and binding to evaluate VMAT2 as a biomarker of beta cell mass.

Similar to past studies [20, 21], despite significant reductions in tracer binding in T1DM patients relative to controls, a greater-than-expected uptake of [^18^F]FP-(+)-DTBZ was measured in body and tail of the patients with T1DM given their almost complete loss of insulin producing cells. Possible reasons for increased uptake in diabetes patients include higher than expected non-specific binding, radioactive metabolites as a confounding source of signal, and pancreatic sites [33, 34] other than β cells expressing VMAT2. Preliminary studies by Naganawa [18] suggest that these sources contribute to less than 15 % of the total tracer binding however. It is interesting to note that a recent nuclear medicine study imaging T1DM patients and controls [35] using an alternative biomarker of BCM, the glucagon-like peptide 1 receptor (GLP-1R) and an In-111 labeled exendin-3 radioligand gave results very similar to those observed for imaging VMAT-2 with [^18^F]FP-(+)-DTBZ. Why two very different radioligands, targeting distinctly different β cell-specific targets, give similar results in both the fractional decrease in pancreatic binding and a residual background uptake of the tracer in the T1DM subjects is not fully understood. Recently, VMAT2 was identified as a late stage regulator of β cell differentiation [36]. It is tempting to speculate that the “background” in our VMAT2 measurements in T1DM may reflect the presence of insulin depleted mature β cells and/or an immature β cell lineage in the pancreas.

It is also possible that this residual VMAT2 resides in ductal and/or exocrine tissue, yet escapes detection by immunohistochemistry because of low-expression levels. To explore this possibility, we revisited the question of VMAT2 expression in the human exocrine pancreas. Normal beta cell mass (based on immunohistochemistry measurements) is routinely quoted at being 1–2 % of the total pancreas ([8, 10] although varies greatly from individual to individual [7]. We found that the average fold difference of expression of VMAT2 message in purified islets relative to whole pancreas was about 40-fold greater and similar to our previously published results [37], about 500-fold greater than purified exocrine tissue. Based on normal beta cell masses ranging from 1 to 2 %, we would expect that the fold difference between islets and whole pancreas would range from 100- to 50-fold, respectively. This possible discrepancy may reflect (a) the fact that the whole pancreas samples used where not from the same donors of the islets or (b) non-islet-associated VMAT2 within the pancreas. However, due to the at least 500-fold greater level of VMAT2 expression in islets relative to exocrine tissue, the contribution of exocrine VMAT2 expression to the total VMAT2 expression is likely to be less than 10 %. Interestingly, even VMAT2 expressed in exocrine tissue might still retain its value as an indirect marker of BCM, as insulin regulates exocrine pancreas growth [38].

Two previous reports call into question the utility of DTBZ-based tracers for PET estimation of pancreatic beta cell mass [39, 40]. The principal conclusion of Erickson et al. [39], based on *in vitro* studies, were that [^18^F]FE-(+)-DTBZ (a tracer similar, but not identical, to [^18^F]FP-(+)-DTBZ used in the current study) bound to human exocrine tissue, in both a non-displaceable and displaceable manner, with a magnitude similar to tracer binding to islet tissue. These authors also reported the presence of tetrabenazine-displaceable, low affinity, but high-capacity tracer binding sites in exocrine tissue homogenates, similar to a report by Tsao, et al. in a study of [^18^F]FP-(+)-DTBZ binding to rat exocrine pancreas tissue homogenates [33]. In a follow-up *in vitro* study, Tsao and colleagues, demonstrated that the low affinity but high-capacity exocrine pancreas tracer binding sites were likely to corresponded to sigma 1 and 2 receptors based on blocking studies with 1,3-Di-o-tolylguanidine (DTG) [34]. Fagerholm, et al. [40], in a study of [^11^C]-(+)-dihydrotetrabenazine (a ligand with less affinity to VMAT2 than [^18^F]FP-(+)-DTBZ) binding to rat and human pancreas tissue, concluded that binding of [^11^C]-(+)-DTBZ and [^3^H]DTBZ in islets of the rat pancreas did not exceed the level of binding in exocrine tissue. Contrary to the studies published by Tsao et al. [33] using [^18^F]FP-(+)-DTBZ, Fagerhom et al. were unable to clearly demonstrate accumulation of either [^11^C] or [^3^H]DTBZ radioactivity in pancreatic islets *in situ* [40].

The above-cited studies [33, 34, 39, 40] based most of their conclusions on *in vitro* studies of tracer binding. The conclusions of these *in vitro* studies (i.e., that DTBZ-based tracers are not suitable for VMAT2 quantification in the pancreas), however, run contrary to the evidence supplied by the more relevant *in vivo* PET studies, performed both in non-human primates (NHP) [5] and humans [18] using the (+) and (−) enantiomers of [^18^F]FP-DTBZ to measure total and non-specific tracer binding, respectively, *in situ*. In the first study, baboons underwent multiple dynamic abdominal and brain PET scans each for 2 h with (+) and (−) enantiomers on separate occasions. Data were analyzed by compartmental models to estimate non-displaceable and specific VMAT2 binding in the striatum, cerebellum, kidney cortex, spleen and pancreas. The non-displaceable distribution volume of [^18^F]FP-(+)-DTBZ in the pancreas, as estimated by the total distribution volume of [^18^F]FP-(−)-DTBZ in the pancreas, was 25 % or less than the specific distribution volume. Naganawa et al. [18], using the same experimental paradigm in human volunteers and NHP, reported that the non-displaceable distribution volume of [^18^F]FP-(+)-DTBZ in the human pancreas, as estimated by the total distribution volume of [^18^F]FP-(−)-DTBZ in the pancreas, was 15 % or less than the specific distribution volume in humans. To assess possible tracer binding to sigma receptors, blocking studies in NHP were performed with fluvoxamine. The extent of displacement by fluvoxamine of [^18^F]FP-(+)-DTBZ binding in the pancreas was negligible *in vivo*. Together, these results suggest that our quantification of pancreatic VMAT2 is reasonably accurate and not overestimated by significant off-target and non-specific tracer binding.

In the cross-sectional PET study with [^18^F]FP-(+)-DTBZ, we attempted to estimate VMAT2 binding in the pancreas of healthy controls and patients with type 1 or 2 diabetes mellitus. As a metric of comparison, we used functional binding capacity, the product of BP_ND_ and volume in the body and tail of the pancreas. We found that VMAT2 binding was decreased in subjects with long-standing type 1 diabetes when compared to healthy controls. More specifically, the functional binding capacity in T1DM patients was 37 % of that observed in healthy controls (*p* = 0.001). In comparison, patients with type 1 diabetes mellitus had on average 3 % insulin secretion capacity on mixed meal tolerance testing when compared to healthy controls (*p* < 0.0001). Our findings are similar to those reported by Normandin et al. [21]. In their study, the authors also assessed [^18^F]FP-(+)-DTBZ PET to compare binding between long-standing T1DM and healthy controls. The Normandin study found that pancreatic BP_ND_ was reduced by 40 % (*p* < 0.01) in patients with long-standing T1DM when compared to healthy individuals matched for age and BMI. C-peptide was undetectable in all patients with T1DM. When pancreatic PET volume was used to calculate a functional binding capacity, total [^18^F]FP-(+)-DTBZ binding in the pancreas was reduced by 59 %. We obtained similar results; functional binding in our study was reduced by 63 %. Normandin and colleagues concluded, as do we, that quantitative evaluation of islet β cell density using VMAT2 as a biomarker can be achieved using [^18^F]FP-(+)-DTBZ.

We also assessed pancreatic [^18^F]FP-(+)-DTBZ uptake in patients with type 2 diabetes mellitus. To our knowledge, this is the first pancreatic PET with [^18^F]FP-(+)-DTBZ study in T2DM. We found no significant difference in functional binding capacity in subjects with T2DM when compared to healthy control (60 ± 20 vs. 102 ± 12 ml, *p* = 0.15). Although there was no significant difference between these two groups, functional binding capacity trended lower to 59 % of controls. This is consistent with autopsy data reported by Butler et al. [41]. In that study of pancreata from lean or obese people with or without diabetes, relative pancreatic β cell volume of diabetic individuals (i.e., insulin dependent) was approximately 50 % of their non-diabetic counterparts. Further studies of T2DM and non-diabetic subjects, with a larger sample size and carefully controlled for adiposity, medical history, treatment, and family history of T2DM, will be needed to ascertain the full utility of pancreatic VMAT2 measurements by PET with [^18^F]FP-(+)-DTBZ in this subset of patients with diabetes.

We report intra-subject reproducibility of [^18^F]FP-(+)-DTBZ in human pancreas. Subjects underwent repeat PET scanning within 1 month. The reliability of the repeat PET scans was good-to-excellent and the mean variability between test and retest scans was about 16 % suggesting that following repeat scanning, the minimal detectable change will be around 18 BP_ND_ × volume units. If we arbitrarily set the mean control FBC (103 units) (Table 3) to represent a BCM of 100 %, at least in the early stages of β cell decline, the MDC of FBC will roughly correspond to the minimal detectable percentage change in BCM (i.e., about 18 %).

## Conclusion

Pancreatic PET [^18^F]FP-(+)-DTBZ uptake is significant reduced in patients with long-standing type 1 diabetes when compared to healthy controls. Intra-subject test retesting showed excellent reliability. These results suggest that longitudinal scanning can be used to track BCM in patients undergoing β cell loss. Such measurements could expedite the assessment of therapeutic efficacy of interventions to onset or treat disease. Further longitudinal studies evaluating the VMAT2 binding in patients with incipient and newly diagnosed type 1 diabetes mellitus should be undertaken.

## Electronic supplementary material

ESM. 1 (PDF 906 kb)
