# Supplementary material for: Cross-sectional and Test-Retest Characterization of PET with [18F]FP-(+)-DTBZ for β Cell Mass Estimates in Diabetes
Source: Mol Imaging Biol. 2015 Sep 14;18:292–301. doi: 10.1007/s11307-015-0888-7 (PMC4783444; doi:10.1007/s11307-015-0888-7)
Supplement: Supplementary file 1 — (PDF 906 kb) [file 11307_2015_888_MOESM1_ESM.pdf]

Crossectional and Test-Retest Characterization of PET with [^{18}F]FP-(+)-DTBZ for β-Cell Mass Estimates in Diabetes.

Journal: Molecular Imaging and Biology

Authors: Matthew J. Freeby[1,2], Patricia Kringas[2], Robin S. Goland[2] , Rudolph L. Leibel[2], Antonella Maffei[3,4],Chaitan Divgi[5], Masanori Ichise[5], and Paul E. Harris[2,4][a].

Abbreviated Title: Beta cell Mass Estimates by PET with [^{18}F]FP-(+)-DTBZ

Manuscript Category: Article

Author Affiliations:

1. Department of Medicine, David Geffen School of Medicine, University of California, Los Angeles, California, 90404.

2. Naomi Berrie Diabetes Center, Columbia University Medical Center, New York, New York 10032

3. Institute of Genetics and Biophysics Adriano Buzzati-Traverso, Consiglio Nazionale delle Ricerche, 80131 Naples, Italy

4. Division of Endocrinology, Department of Medicine, Columbia University Medical Center, New York, New York 10032

5. Division of Nuclear Medicine and Kreitchman PET Center, Department of Radiology, Columbia University Medical Center, New York, New York 10032

[a] Address correspondence to Paul E. Harris; email: peh1@cumc.columbia.edu

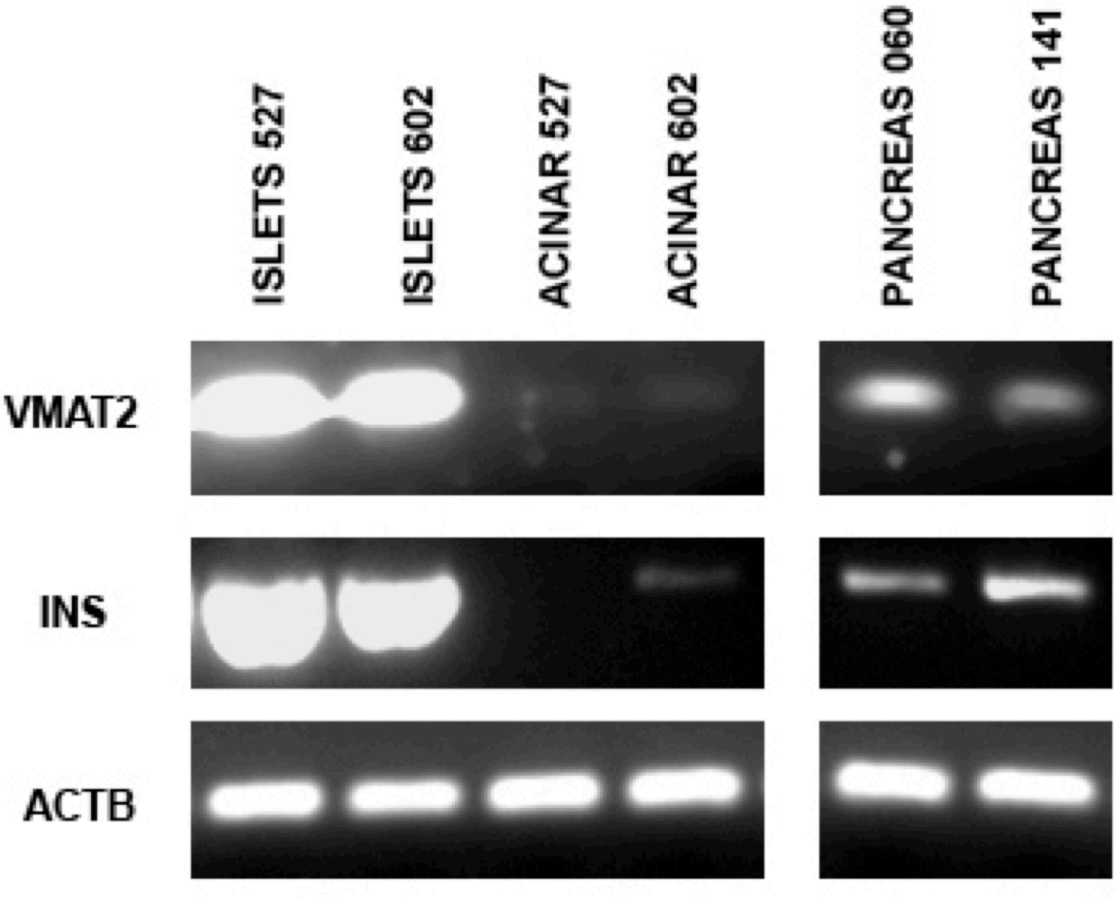

Supplemental Figure 1. Relative expression of VMAT2, Insulin, and Beta-actin mRNA in pair matched islets and exocrine pancreas tissue and third party whole pancreas. A RT-PCR analysis with primers specific for (top row) VMAT2, (middle row) Insulin, and beta-actin (bottom row). From left to right , Lane 1 – purified cadaveric islets (donor 527), Lane 2- purified cadaveric islets (donor 602), Lane 3 – purified exocrine tissue (donor 527), Lane 4- purified exocrine tissue (donor 602), Lane 5 – commercial whole pancreas total RNA (clonetech) and Lane 6 – commercial whole pancreas total RNA (agilent).
